# Effects of Quinoa Secondary Metabolites on In Vitro Fermentation and Gas Production

**DOI:** 10.3390/ani15111522

**Published:** 2025-05-23

**Authors:** Junfeng Ge, Yindi Yang, Hao Lu, Bo Wang, Hongjin Yang, Shanli Guo

**Affiliations:** 1 College of Grassland Sciences, Qingdao Agricultural University, Qingdao 266109, China; gejunfengqau@sina.com (J.G.); m15101815208@163.com (Y.Y.); luhao199901@163.com (H.L.); wangbo@qau.edu.cn (B.W.); 17660249181@163.com (H.Y.); 2 Key Laboratory of National Forestry and Grassland Administration on Grassland Resources and Ecology in the Yellow River Delta, Qingdao Agricultural University, Qingdao 266109, China; 3 College of Life Sciences, Yantai University, Yantai 264005, China

**Keywords:** quinoa lines, methane reduction, secondary metabolites, in vitro fermentation, new feed sources

## Abstract

Livestock methane emissions are a significant source of greenhouse gases. The aim of this study was to investigate the secondary metabolites of different strains of silage quinoa and their impact on methane emissions from livestock farming. In this study, we evaluated the chemical composition, fermentation quality, secondary metabolite content, and in vitro gas production of eight quinoa lines, 093, 137, 231, 238, 565, 666, 770, and 811, grown in saline and alkaline areas of the Yellow River Delta. The results showed that crude protein, EE, and crude ash content ranged from 8.84% to 10.69%, 1.98% to 2.38%, and 17.00% to 23.14%, respectively. The acidic and neutral detergent fiber content of these eight quinoa varieties ranged from 49.31% to 61.91% and 33.29% to 37.31%, respectively. Line 093 had the highest total saponin content, while Line 231 exhibited the highest flavonoid content. Methane yield was significantly and negatively correlated with tannin, saponin, and flavonoid content, whereas carbon dioxide yield showed a positive correlation with saponin and flavonoid content. Among all lines, 770 and 811 demonstrated the lowest methane production, indicating strong in vitro inhibition of methanogenesis. These findings suggest that feeding quinoa silage to ruminants has the potential to reduce greenhouse gas emissions.

## Simple Summary

Agricultural greenhouse gas emissions constitute a significant portion of global emissions, with methane produced by rumen fermentation in ruminant livestock being particularly notable. This study investigates the feeding effects of the emerging forage crop quinoa, moving away from traditional additive-based rumen regulation methods. Instead, it utilizes secondary metabolites abundant in quinoa, such as saponins and tannins, to modulate rumen activity. The research verifies the impact of quinoa on greenhouse gas emissions and identifies two quinoa varieties suitable for use as forage among several tested. These findings offer new insights into the exploration of novel forages and provide methods for reducing greenhouse gas emissions in livestock production.

## 1. Introduction

Greenhouse gases (GHGs) are a major contributor to global warming by absorbing infrared radiation and trapping heat in the Earth’s atmosphere [1]. While reducing emissions from energy sources has been a primary focus, there is also significant potential for emissions reductions from agriculture [2]. Agricultural production processes account for approximately 17% of global GHG emissions, with livestock contributing 14.5%. Methane (CH₄) is a key GHG produced by ruminants, representing about 40% of their total GHG emissions and resulting in a feed energy loss of 2–12% [3]. Consequently, reducing methane emissions is not only crucial for mitigating global warming but also for improving feed utilization efficiency [4]. Feed composition plays a crucial role in methane emissions, with high-fiber feeds generally increasing methane production [5]. Inhibition of methane production through natural plant secondary metabolites has become an important research topic, as these compounds can decrease emissions by either inhibiting rumen protozoa or exerting toxic effects on methanogenic bacteria [6,7,8,9]. Therefore, exploring new feeds to decrease methane emissions has become a hot topic of research.

Quinoa (*Chenopodium quinoa* Willd) is an annual plant from the quinoa family, widely cultivated for its nutritious seeds [10]. With a protein content exceeding 10%, quinoa is considered a high-quality feed alternative [11]. Furthermore, quinoa is rich in secondary metabolites, such as saponins, flavonoids, and tannins, which possess various health-promoting effects, including antioxidant and anti-inflammatory properties [12]. These metabolites also have the potential to decrease methane production during rumen fermentation [13,14]. The concentration of secondary metabolites in quinoa can vary from 0.01% to 4.65% of dry matter, depending on the variety and environmental conditions [15]. Some studies have demonstrated that quinoa cultivation can lower greenhouse gas emissions, contributing to the development of more sustainable agricultural systems [16].

Historically, quinoa was used by indigenous people in South America as livestock feed, with the straw also serving as fodder [17]. Recent studies have found that replacing triticale hay with up to 45% quinoa hay does not adversely affect rumen fermentation [18]. Similarly, adding quinoa seeds to broiler diets has been shown to improve growth performance and health [19], while feeding quinoa seeds to lambs enhances immunity, decreases blood cholesterol levels, and improves meat quality [20]. These findings suggest that quinoa, as a feed ingredient, is not only nutritious but also has the potential to decrease methane emissions.

Methanogenesis is an intrinsic process of rumen fermentation, and inhibiting it presents a challenge. While chemical inhibitors are effective, they are often toxic to animals or negatively affect rumen fermentation [21]. Therefore, exploring natural plant secondary metabolites, such as those found in quinoa, as a means of methane mitigation is essential.

The objective of this study was to screen quinoa lines with potential for greenhouse gas inhibition, assess the nutrient composition, fermentation quality, and secondary metabolite content of quinoa from different lines, and examine the effects of secondary metabolites on in vitro fermentation and methane production. By analyzing the relationship between fermentation quality, secondary metabolites and gas production, this study verified the potential role of using quinoa silage fed to ruminants in reducing greenhouse gas emissions.

## 2. Materials and Methods

### 2.1. Overview of the Test Site

The experimental quinoa planting site is located in the Yellow River Delta Agricultural Hi-Tech Industrial Demonstration Zone, Guangrao County, Dongying City, Shandong Province, at a geographic location of 118.652176° E longitude and 37.318027° N latitude, with an elevation of 5.3 m. Dongying City belongs to the continental climate of the warm temperate zone with a semi-moist monsoon type. The annual mean temperature is 13.3 °C, and the region experiences uneven rainfall, with droughts during winter, spring, and late autumn. Precipitation is mostly concentrated in July and August, accounting for 52.74% of the total annual rainfall. The average annual evapotranspiration is about 1.5 mm, and the average annual evaporation is 1885 mm. The area receives sufficient sunlight, with an average annual total radiation of 533.0 J·cm^−2^. Soil pH is 8.8, soil organic matter content is 14.4 g/kg, and soil total nitrogen content is 0.8 g/kg.

To minimize the influence of environmental factors on the experimental results, the following variables were controlled during the experimental process: temperature and humidity were kept constant, and all treatments and measurements were conducted under the same equipment conditions.

### 2.2. Plant Material

Eight quinoa lines from different countries (e.g., Chile, Argentina, and the United States) were selected for this study (Table 1), as they are representative in terms of nutrient composition, adaptability, and yield potential. The eight quinoa lines selected, although the germplasm resources were from other regions, were not affected by genetic or environmental factors in terms of changes in secondary metabolites because they were grown on saline soils in the Yellow River Delta. These lines were chosen for their performance under different climatic conditions and their potential silage value. They are more suitable for the environment of the Yellow River Delta region, with a higher nutrient content. The purpose of selecting these lines was to evaluate their potential for methane reduction in ruminants and their suitability for cultivation in marginal lands.

A large harvester was used to harvest the quinoa, and samples were taken during the quinoa filling period as pre-silage samples. All test materials were dried to a moisture content of about 70% and then chopped to 2.0 cm using a large chopper for collection. The material was sprayed with 1:1000 lactic acid bacteria (Shanxi Guanchen Biotechnology Co., Ltd., Xi’an, China) and wrapped in silage with a large silage wrapping machine for 120 d of fermentation. Samples were taken before and after silage to determine DM, CP, EE, ADF, NDF, and Ash. These components are determined on a dry matter basis. The dry matter content of quinoa before silage ranged from 17.33% to 22.87%, protein from 8.04% to 13.84%, crude fat from 1.76% to 3.28%, crude ash from 15.62% to 20.39%, NDF from 43.11% to 52.58%, and ADF from 28.47% to 35.94%. After fermentation, the dry matter content ranged from 21.64% to 34.17%, protein from 8.84% to 10.69%, crude fat from 1.98% to 2.38%, crude ash from 17% to 23.14%, NDF from 49.31% to 61.91%, and ADF from 33.29% to 37.31%.

Another 20 g of silage samples were mixed with 180 mL of distilled water, shaken for 10 min, filtered through four layers of gauze, and then filtered through qualitative filter paper to obtain the silage extract. Part of the extract was centrifuged at 12,000 rpm for 10 min, passed through a 0.22 μm filter membrane for the determination of organic acids, and used for subsequent determination of fermentation quality and chemical analysis. The fermentation and chemical quality tests, as well as the in vitro fermentation quality, were repeated three times.

### 2.3. In Vitro Fermentation

The rumen fluid used in this experiment was collected from animal experiments approved by the Ethics Committee, and all experimental procedures complied with the regulations of the Ethics Committee for Animal Experiments of the Beijing Animal Husbandry and Veterinary Medicine Institute. Rumen fluid used for in vitro fermentation was collected from three fistulated dairy cows in mid-lactation at the Changping Experimental Base of the Beijing Animal Husbandry and Veterinary Medicine Institute (BAHVRI). The cows were fed a total mixed ration (TMR) formulated to meet both maintenance and production nutritional requirements. Sampling was conducted prior to the morning feeding to ensure consistent microbial activity in the rumen fluid. The in vitro fermentation experiment was repeated three times. The rumen fluid collection was approved by the relevant authorities. On the day of the experiment, rumen fluid was collected before morning feeding and transferred to a thermos flask filled with carbon dioxide, preheated to 39 °C to maintain an anaerobic environment, and brought back to the laboratory quickly. The fluid was then filtered through four layers of gauze [22].

An artificial rumen buffer was prepared according to Menke et al. Menke’s medium was prepared in the ratio specified in Table 2, and anaerobic conditions were maintained using a reducing agent in the medium [23]. Three fermentation bottles were taken from each treatment group for replication of the experiment, and the medium was then thoroughly mixed with the insulated rumen fluid from the three cows previously obtained at a ratio of 2:1 for each treatment group. A total of 150 mL of the mixed artificial rumen culture solution was dispensed into each fermentation flask, which was immediately sealed tightly and placed in a constant-temperature gas bath shaker (THZ-C-1, Taicang Haocheng Experimental Instrument Manufacturing Co., Ltd., Taicang, China) at 39 °C, shaking at a rate of 80 r/min to carry out in vitro fermentation.

Gas production was determined using the ANKOM RFS gas production measurement system (ANKOM Technology Corp., Macedon, NY, USA). The device features an automatic wireless GP module with a fermentation flask containing a pressure sensor. The substrate was dried in an oven at 65 °C until a constant weight was achieved, then crushed through a 40-mesh sieve for use. Two grams of fermentation substrate were accurately weighed and placed into a filter bag (organic basis). The filter bag was then placed into a fermentation flask to determine the cumulative gas production (GP) at standard pressure and temperature, which was converted to volume. The gas production in the empty flask (empty flask corrected GP) was subtracted, and readings were recorded every hour to obtain the net GP. The gas produced from each incubation was collected through the sampling holes in each module for subsequent testing.

Before analysis, the GC was calibrated with standard gas mixtures containing known concentrations of CH_4_, CO_2_, and other relevant gases. The gas composition was quantified by comparing sample retention times and peak areas with those of the standards. This methodology ensures precise differentiation and quantification of individual gas components, addressing potential concerns regarding CO_2_ production under anaerobic conditions [24].

According to the National Environmental Protection Standard of the People’s Republic of China (HJ38-2017) [25], the content of each component in the gas was determined using gas chromatography. The analysis was carried out with a gas chromatograph (model GC112A, Shanghai Yidian Analytical Instruments Co., Ltd., Shanghai, China) equipped with a 5A stainless steel column (Φ3 mm × 3 m, with a stretcher 60–80 mesh Chromosorb) and a Tbx-01 stainless steel column (Φ3 mm × 1 m, 60–80 mesh Chromosorb). One column was used for the determination of methane, oxygen, and nitrogen, while the other was used solely for carbon dioxide determination. The chromatographic conditions were as follows: column temperature of 100 °C, TCD detector temperature of 100 °C, inlet temperature of 100 °C, and an injection volume of 1 mL. The carrier gas was high-purity argon with a flow rate of 30 mL/min and a pressure of 0.4 MPa. The specific content of each gas component was obtained by multiplying the percentage of each gas component by the GP [26].

### 2.4. Chemical Analyses

The pre-silage and silage samples were dried at 65 °C for 48 h to a constant weight to calculate the moisture content. The dried plants were then crushed to determine the crude fat content using an automatic fat analyser (ANKOM XT10i), protein content using an elemental analyzer (Elementar Analysensysteme GmbH, Langenselbold, Germany), neutral detergent fibers (NDF), and acid detergent fibers (ADF) using an automatic cellulose analyzer (ANKOM 2000i, Macedon, NY, USA), and crude ash content using the high-temperature oven ashing method (Thermo Fisher Scientific Inc., Waltham, MA, USA) [27,28]. Determination of ammoniacal nitrogen was made by sodium hypochlorite-phenol reagent spectrophotometry [29]. Saponins, tannic acid and flavonoids were determined using a kit produced by Suzhou Keming Biological Products Co., Ltd. (BioNano Park, Suzhou, China) and analyzed using a spectrophotometer (Agilent Technologies Inc., Santa Clara, CA, USA). The extraction of saponins was performed by adding 0.05 g of dry plant sample to 1 mL of methanol containing 1% ammonia, ultrasonic extraction for 1 h, then centrifugation at 8000 r/min for 10 min to achieve the supernatant; vanillin-perchloric acid colorimetry was then used to achieve an absorbance value of 589 nm and a difference with the blank control group, and then it was substituted into the regression curve to achieve the total saponin content [30,31]. Tannins react with phosphomolybdic acid in an alkaline environment to form a blue compound with a maximum absorption peak at 760 nm [32,33]. For the determination of flavonoids, in alkaline nitrite solution, flavonoids and aluminum ions formed a red complex with a characteristic absorption peak at 510 nm. The absorbance value of the sample extract at 510 nm can be measured to calculate the flavonoid content of the sample [34]. Because of different determination methods, saponins and flavonoids are determined on the basis of dry matter and tannins are determined on the basis of the original sample.

Chromatographic-grade acetonitrile was used as phase A. A 20 mmol/L solution of analytically pure potassium dihydrogen phosphate was used to adjust the pH to 2.9 with phosphoric acid and passed through a 0.45 μm filtration membrane as phase B. The column temperature was set to 30 °C, the detection wavelength was 220 nm, and the flow rate was 1 mL/min. A to B ratio of 2:8, isocratic elution. Standard curves for lactic acid, acetic acid, propionic acid, and butyric acid were prepared using the respective standards. The results were analyzed using Chromeleon™ Dionex version 7.2.10, and the peaks were integrated based on the standard curves. The pH of the silage extracts was determined using a Rembrandt pH meter.

### 2.5. Statistical Analyses

The experimental data were recorded using Excel 2019 and analyzed with SPSS (version 24.0, IBM, Armonk, NY, USA) for integration and ANOVA testing. A one-way analysis of variance (ANOVA) was conducted for each index across different strains, and multiple comparisons were performed using the LSD method. Statistical significance was set at a *p*-value of <0.05. The results are presented as “mean ± SEM” and were plotted using Origin 2021.

The gas production scale readings were recorded, and the gas production was corrected by a blank control, calculated using the following formula:

$${GP}_{t}=200\times (V_{t}-V_{0})/w$$

where *GP_t_* is the gas production (mL) of the sample at the time, *V_t_* is the scale reading of the incubation tube after *t* hours of fermentation, *V*_0_ is the scale reading of the blank incubation tube at the beginning of the incubation, and *w* is the weight of the sample in dry matter.

The organic matter digestibility (OMD), metabolizable energy (ME), and net energy for lactation (NE_L_) of silage quinoa in the experiment were obtained using the following formulas [35,36]:

$$OMD\left(\%\right)=14.88+0.8893\times GP+0.448\times CP+0.651\times Ash$$

$$ME\left(\frac{MJ}{kg}DM\right)=2.20+0.136\times GP+0.057\times CP+0.002859\times EE$$

$${NE}_{L}(\frac{MJ}{kg}DM)=0.101\times GP+0.051\times CP+0.11\times {EE}^{2}$$

where GP is the 24 h net gas production (mL/200 mg DM), CP is crude protein (%), EE is ether extract (%), and Ash is crude ash (%).

## 3. Results

### 3.1. Fermentation Quality

As shown in Table 3, the pH of silage quinoa extracts ranged from 4.44 to 4.89, with line 811 having a significantly higher pH than the other lines (*p* < 0.01), while line 666 had a significantly lower pH than the other lines except for line 231 (*p* < 0.01). The ammoniacal nitrogen content of different quinoa silage lines ranged from 19.28 g/kg FM to 40.14 g/kg FM, with line 666 having the lowest ammoniacal nitrogen (19.28 g/kg FM) and lactic acid content (4.41 g/kg FM). The highest lactic acid content was found in line 238 (19.65 g/kg FM), and the highest acetic acid content ranged from 133.53 g/kg FM to 473.02 g/kg FM, with line 231 having the highest acetic acid content (473.02 g/kg FM). Propionic acid and butyric acid were not detected.

### 3.2. Secondary Metabolites

As shown in Table 4, the total saponin content was significantly different (*p* < 0.01) among the different lines, ranging from 2.71 to 5.6 g/kg DM. Line No. 093 had the highest saponin content (5.6 g/kg DM), while No. 565 had the lowest (2.71 g/kg DM). Tannin and flavonoid content ranged from 4.53 to 5.79 g/kg DM and 6.36 to 10.43 g/kg DM, respectively. Line No. 231 had significantly higher flavonoid content than the other lines (10.43 g/kg DM, *p* < 0.01).

### 3.3. In Vitro Fermentation Production

**Figure 1:**
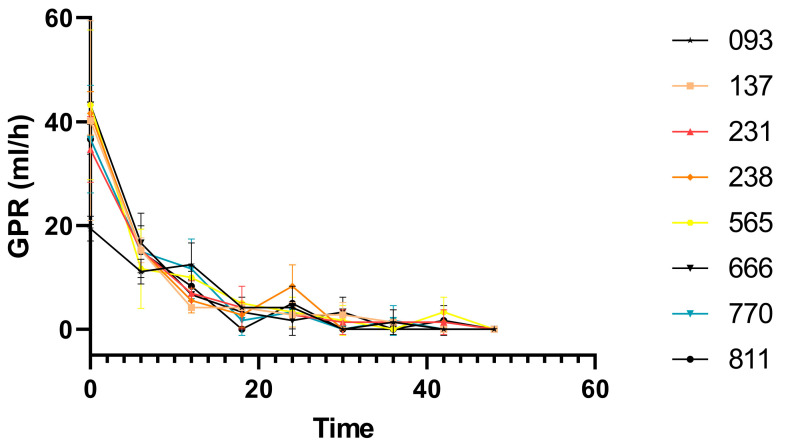
Temporal gas production rates of silage quinoa lines. GPR: gas production rate.

As shown in Figure 1, the rate of silage gas production for each quinoa strain decreased over time, gradually approaching zero from the 32nd h onward. By the 48th h, almost all strains had a rate of 0.0. As indicated in Table 5, in vitro fermentation gas production (GP) showed significant differences among different silage quinoa lines, ranging from 116.99 mL/g to 132.97 mL/g. Silage quinoa No. 666 produced the highest GP of 132.97 mL/g after 48 h of in vitro fermentation and was significantly different (*p* < 0.01) from the other lines. In contrast, silage quinoa No. 093 produced the lowest gas production at 116.99 mL/g, which differed significantly (*p* < 0.01) from No. 666.

### 3.4. Gas Composition

As shown in Table 6, the 48 h methane (CH_4_) yield ranged from 7.99 to 14.03 mL/g, with No. 238 having the highest yield at 14.03 mL/g, significantly higher than the other lines (*p* < 0.01), and No. 770 having the lowest yield at 7.99 mL/g. The 48 h carbon dioxide (CO_2_) yield ranged from 22.78 to 32.93 mL/g, with No. 238 again being the highest at 32.93 mL/g, and No. 811 being the lowest at 22.78 mL/g. Other gases, such as hydrogen (H_2_), were not detected.

### 3.5. Digestibility and Energy Value

As shown in Table 7, the organic matter digestibility (OMD) of different strains of silage quinoa ranged from 52.20% to 55.59%, with No. 666 being the highest (55.59%) and No. 137 being the lowest (52.20%), showing significant differences (*p* < 0.01). The metabolizable energy (ME) ranged from 5.97 to 6.34 MJ/kg DM, with No. 666 having the highest value (6.34 MJ/kg DM) and No. 093 the lowest (5.97 MJ/kg DM). The net energy of lactation (NE_L_) ranged from 3.12 to 3.40 MJ/kg DM, with No. 666 being the highest and No. 093 the lowest, with significant differences (*p* < 0.01). These results indicate that No. 666 performed best in terms of digestibility and energy value.

### 3.6. Correlation

**Figure 2:**
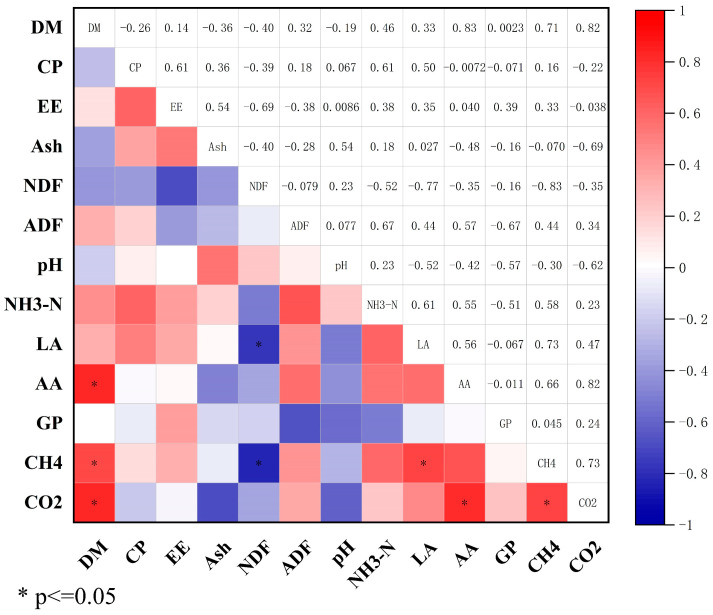
Correlation between chemical composition, fermentation quality and gas production. Red indicates a positive correlation, while blue represents a negative correlation. * *p* < 0.05.

The results of correlation analysis (Figure 2) showed that the dry matter content was significantly and positively correlated (*p* < 0.05) with carbon dioxide (CO_2_), methane (CH_4_), and acetic acid (AA) content, with correlation coefficients of 0.82, 0.71, and 0.83, respectively. The NDF content was significantly negatively correlated (−0.73, *p* < 0.05) with methane yield. The ADF content was significantly positively correlated with acetic acid (0.73, *p* < 0.05), and the lactic acid content was positively correlated with methane (0.73, *p* < 0.05). There was also a significant positive correlation between methane and carbon dioxide (0.73, *p* < 0.05).

### 3.7. Principal Component Analysis

**Figure 3:**
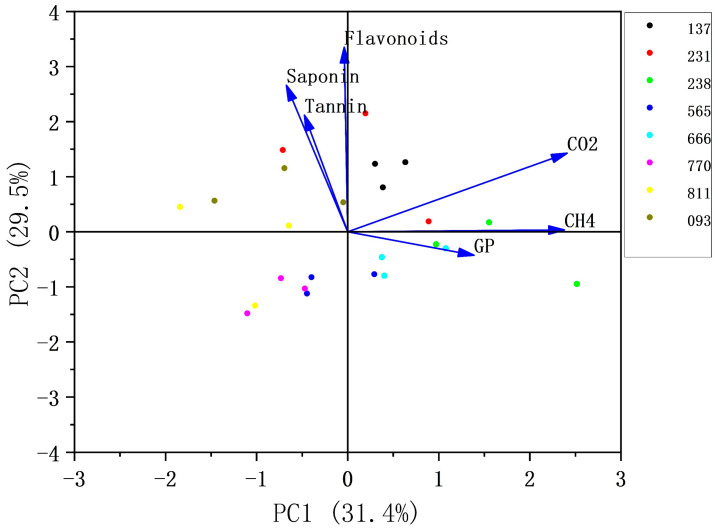
Principal component analysis between nutrient composition and gas production. Circles represent the distribution of different strains, while arrows indicate the relationships between different substances.

Principal component analysis (PCA) was performed using six variables, including saponins, tannins, flavonoids, CO_2_, CH_4_, and total gas production. The first principal component (PC1, 31.4%) primarily captured variation in gas production and the composition of different gases, whereas the second principal component (PC2, 29.5%) mainly represented differences in the secondary metabolite content. Gas production and secondary metabolites of eight lines of silage quinoa were analyzed using principal component analysis (PCA, Figure 3). The PCA revealed that CO_2_ and CH_4_ were located in the same quadrant, with lines 238 and 137 clustered in the positive direction. Gas production (GP) was negatively correlated with tannins, saponins, and flavonoids, while methane (CH_4_) was negatively correlated with these secondary metabolites. Additionally, CO_2_ was positively correlated with saponins and flavonoids. These results highlight the relationships between gas production, fermentation quality, and the secondary metabolite content in silage quinoa.

## 4. Discussion

Chemical composition is a crucial indicator for evaluating the nutritional value of forages, with the protein content being a key factor in determining forage quality. In this study, the crude protein content of quinoa ranged from 8% to 14%, while the NDF content varied from 43% to 52%, and the ADF content ranged from 29% to 36%. In comparison, the protein content in the study by Ebeid et al. was lower than 18.6%, while NDF was approximately 46.4%, and ADF was higher than 24.9% [18]. Similarly, the protein content in Mustafa et al.’s study was 15.31%, and the ash content exceeded 6.21% [19]. Research on different quinoa varieties has shown significant differences in protein content based on variety, cultivation year, and environmental conditions [37]. The lower protein content observed in this study may be attributed to the combination of these factors.

Silage is an effective method for preserving quinoa’s nutrients and addressing storage challenges. Silage fermentation in this study did not produce propionic or butyric acids (Table 3), indicating that quinoa forage can be well preserved. However, the low lactic acid bacteria count and high acetic acid content may be attributed to the extended silage duration, insufficient dry matter content, and lack of added energy substances, which hinder the growth of lactic acid bacteria and lead to acetic acid-dominated fermentation. The crude fat content showed a slight decrease with extended fermentation time, possibly due to lipid degradation by microorganisms or storage-related losses. Longer fermentation times also positively impacted digestibility [38]. Dong et al. found that direct silage of freshly cut quinoa tends to promote acetic acid-type fermentation, but the addition of molasses can effectively enhance lactic acid fermentation [39]. Ertekin et al. demonstrated that quinoa harvested at the wax-ripening stage exhibited better aerobic stability and produced higher-quality silage [40]. Fang et al. showed that adding molasses and lactic acid bacterial agents improves the efficiency of lactic acid fermentation in silage [41]. However, excessive molasses may improve dry matter quality but decrease in vitro digestibility [42]. Furthermore, Suárez et al. observed a decrease in protein content after 120 d of fermentation compared to short-term silage [43]. High-quality silage requires adequate energy supplementation, which can be achieved by co-silaging with high-energy forages or adding molasses.

Secondary metabolites significantly impact rumen fermentation and methanogenesis. Saponins added to feed can decrease rumen protozoa and methanogenic bacteria [44]. The saponin content in quinoa in this study ranged from 2.7 to 5.6 g/kg DM, significantly lower than the 11% to 22% found in quinoa seed coats [45]. These secondary metabolites do not adversely affect animal health but are sufficient to regulate rumen fermentation. Saponins and tannins decrease methanogenesis by binding to rumen proteins and disrupting the membrane structure of methanogens and protozoa [46]. Studies have shown that saponin addition can significantly decrease methane emissions. For instance, Goel et al. found that Sesbania saponins decreased methanogen populations by 78% [47], and Aderao et al. showed that plant saponin additives decreased methane production by 10% to 25% [48]. Secondary metabolites, such as polyphenols and flavonoids, are positively correlated with methane suppression potential [49]. These compounds have excellent antioxidant and antimicrobial properties, improving rumen digestibility, reducing methane emissions, and enhancing milk production [50,51]. Therefore, quinoa’s secondary metabolites not only optimize fermentation but also increase its overall value as forage. In this study, quinoa lines were cultivated in the saline region of the Yellow River Delta, where soil salinity and pH induce stress responses in plants. As part of the plant’s adaptive mechanism, such stress conditions enhance the synthesis of secondary metabolites, including saponins, flavonoids and tannins. Mild salt stress significantly increased phenolic as well as flavonoid content in quinoa, and similar findings have been reported [52].

The total gas production from in vitro fermentation of quinoa ranged from 116.99 to 132.97 mL/g DM, with methane production ranging from 8 to 14 mL/g DM. The low methane production may be due to the effect of quinoa’s secondary metabolites, such as saponins and tannins, which inhibit methanogenic bacterial activity. In addition, Kuvera et al. demonstrated that plants rich in condensed tannins and saponins decreased methanogenesis by 10% to 25%, aligning with the findings of the present study [53]. Aderao et al. found that various plants used in an in vitro fermentation assay showed a total gas production of 126 to 151 mL/g DM and methane production of 12.7 to 28.4 mL/g DM. In comparison, quinoa exhibited a more significant methane inhibition in this study [48]. Kozlowska et al. observed that saponin-rich alfalfa varieties decreased methane production without negatively affecting fermentation parameters, which is consistent with this study [54].

When assessing quinoa silage’s nutritional value, organic matter digestibility and energy content are key factors. Mahmoud’s study showed that organic matter digestibility in several herbaceous plants, including grasses and legumes, ranged from 33.66% to 64.11%, which is similar to the present study’s results [55]. Metabolizable energy (ME) is a critical feeding criterion, helping to accurately balance animal rations [56]. Quintero-Anzueta et al. found that the ME of leguminous forages and grasses ranged from 5.9 to 8.5 MJ/kg DM, slightly higher than the present study’s findings [57]. Overall, the digestibility and energy values of quinoa silage are comparable to those of traditional feeds, suggesting its potential application, especially in areas where traditional feed resources are scarce or unsustainable.

Pal et al. reported that the methane yield was negatively correlated with crude protein (CP), ether extract, and non-fibrous carbohydrates (NFC), while positively correlated with neutral detergent fiber (NDF) and acid detergent fiber (ADF) [58]. However, in the present study, methane production was significantly and positively correlated with dry matter and carbon dioxide production, but showed no significant correlation with NDF and ADF. Additionally, Hariadi et al. found a negative correlation between methane production and tannin content after 48 h of in vitro incubation, which aligns with this study’s results [59]. Similarly, Angeles-Mayorga et al. demonstrated that condensed tannins (CT) were negatively correlated with methane yield, further supporting the present study’s findings [60]. Although Angeles-Mayorga et al. did not find a significant correlation between saponin content and methane yield, the present study found a negative correlation between saponins and methane yield. This result contrasts with Trotta et al.’s conclusion that saponins only increase propionic acid proportion without affecting methane production, suggesting that experimental conditions influence saponin mechanisms [61].

It is possible that the combined presence of saponins and tannins contributed to the observed decrease in methane production. Jensen et al. used principal component analysis (PCA) on metabolomics data and found that specific flavonoids from industrial hemp could suppress methane in rumen fermentation, consistent with this study’s findings [62]. Phenolic compounds, such as tannins, decrease methane production by forming complexes with proteins and carbohydrates, aligning with this study’s conclusions [60]. Moreover, Jayanegara et al. emphasized that PCA can classify forages and identify those with high nutritional quality and low methane production [63]. In this study, lines numbered 770, 811, and 093 were located in the negative region of PC1, suggesting they have higher methane suppression potential. Among them, lines 770 and 811 not only demonstrated better methane suppression but also exhibited higher organic matter digestibility and metabolizable energy, making them more suitable for use as high-quality forage.

This study has several limitations. The quinoa lines analyzed were cultivated under specific saline–alkaline conditions in the Yellow River Delta, which may limit the applicability of the findings to other regions with different environmental factors. Variations in soil properties, climate, and management practices could influence secondary metabolite composition and their effects on methane production. Additionally, genetic variability among quinoa lines could contribute to inconsistent results under different growth conditions. Future studies should explore a wider range of lines across diverse environments to validate these findings. Finally, the in vitro nature of this study, while valuable for controlled analyses, may not fully reflect in vivo conditions. Factors such as feed intake behavior, digestion kinetics, and rumen microbial interactions were not accounted for. Further in vivo trials are necessary to confirm the practical effects of quinoa silage on methane emissions and ruminant performance.

## 5. Conclusions

This study highlights the role of secondary metabolites in quinoa for reducing methane emissions and improving feed quality. Quinoa lines 770 and 811, rich in saponins, tannins, and flavonoids, showed significant inhibition of methane production in in vitro fermentation. These results stress the importance of selecting quinoa lines with optimal secondary metabolites for ruminant feeding strategies. Additionally, quinoa silage demonstrated good digestibility and energy values, highlighting its potential as a sustainable, eco-friendly feed resource.

Cultivating these quinoa lines in saline–alkaline soils offers the dual benefit of improving feed quality and reducing greenhouse gas emissions, aligning with climate change mitigation goals. Future research should focus on in vivo trials and the economic feasibility of incorporating quinoa into commercial feed systems for broader adoption. This should include assessments of animal performance and comprehensive cost–benefit analyses under real-life farming conditions.

## Figures and Tables

**Table 1:** Quinoa silage origin and nutritional composition (%).

Code	CP	EE	Ash	NDF	ADF	Source
093	10.11	2.15	19.43	59.47	34.97	Chile
137	9.50	1.98	17.00	61.91	36.06	Chile
231	10.17	2.33	19.74	57.73	35.24	Chile
238	10.18	2.25	20.58	49.31	37.31	Chile
565	10.69	2.14	18.90	62.53	36.66	Argentina
666	8.84	2.38	20.13	51.74	33.96	United States
770	10.32	2.10	21.20	59.42	34.84	Argentina
811	9.93	2.30	23.14	58.02	33.29	United States

Note: DM: DM stands for dry matter, CP represents crude protein, EE refers to ether extract, ADF indicates acid detergent fiber, NDF stands for neutral detergent fiber, and Ash refers to crude ash. All of the above ingredients are measured on a dry matter basis.

**Table 2:** Composition of Menke’s medium.

Stock Solution	Volume (mL)
Buffer (B solution)	208.1
Macroelement solution (C liquid)	208.1
Trace element solution (solution A)	0.1
0.1% resazurin solution (D solution)	1
Deionized water	520.2
Reductant solution (E liquid)	62.4

Note: The Menke culture medium consists of a buffer solution (B solution) for pH stabilization, a microelement solution (C liquid) providing essential minerals, a trace element solution (solution A) supplying micronutrients, 0.1% resazurin solution (D solution) as a redox indicator, deionized water as the solvent, and a reductant solution (E liquid) to maintain anaerobic conditions.

**Table 3:** Fermentation quality of eight silage quinoa lines.

Varieties	pH	NH_3_-N (g/kg FM)	LA (g/kg FM)	AA (g/kg FM)	PA	BA
093	4.63 ± 0.03 b	32.46 ± 7.6 ab	11.46 ± 4.73 ab	230.84 ± 78.38 ab	ND	ND
137	4.59 ± 0.02 bc	23.19 ± 8.25 ab	4.15 ± 1.13 b	364.54 ± 92.69 ab	ND	ND
231	4.5 ± 0.01 de	32.32 ± 9.68 ab	12.75 ± 5.47 ab	473.02 ± 190.68 a	ND	ND
238	4.53 ± 0.01 cd	40.14 ± 5.73 a	19.65 ± 2.14 a	463.68 ± 78.85 a	ND	ND
565	4.55 ± 0.02 cd	30.29 ± 7.56 ab	11 ± 3.85 ab	275.96 ± 58.06 ab	ND	ND
666	4.44 ± 0.01 e	19.28 ± 1.26 b	9.43 ± 2.4 b	163.36 ± 30.11 b	ND	ND
770	4.57 ± 0.01 bc	21.16 ± 2.13 ab	9.87 ± 0.48 b	161.99 ± 11.79 b	ND	ND
811	4.89 ± 0.02 a	31.88 ± 5.12 ab	6.17 ± 1.52 b	133.53 ± 36.66 b	ND	ND
SEM	0.03	2.37	1.36	37.3	-	-
*p* value	<0.01	0.38	0.09	0.08	-	-

Note: Different letters represent significant differences (*p* < 0.05); three replicates were taken for each strain, where pH: Potential of Hydrogen, NH_3_-N: ammoniacal nitrogen, LA: lactic acid, AA: acetic acid, PA: propionic acid, BA: butyric acid. ND: indicates not detected. FM: fresh matter.

**Table 4:** Secondary metabolites of eight silage quinoa lines.

Varieties	Total Saponins (g/kg DM)	Tannin (g/kg FM)	Flavonoid (g/kg DM)
093	5.6 ± 0.24 a	5.65 ± 0.74 a	8.47 ± 0.2 b
137	5.05 ± 0.22 ab	5.56 ± 0.58 a	9.65 ± 0.32 a
231	5.15 ± 0.26 ab	5.59 ± 0.99 a	10.43 ± 0.55 a
238	4.5 ± 0.64 abc	4.55 ± 0.17 a	6.94 ± 0.34 cd
565	2.71 ± 0.03 d	5.79 ± 0.32 a	7.24 ± 0.07 cd
666	3.68 ± 0.19 cd	5.33 ± 0.16 a	7.74 ± 0.3 bc
770	4.26 ± 0.14 bc	4.53 ± 0.5 a	6.36 ± 0.25 d
811	5.09 ± 0.45 ab	5.15 ± 0.75 a	7.6 ± 0.43 bc
SEM	0.21	0.20	0.29
*p* value	<0.01	0.69	<0.01

Note: Different letters represent significant differences (*p* < 0.05); three replicates were taken for each experiment. Where DM: dry matter, FM: Fresh matter.

**Table 5:** Total gas production of each silage quinoa line at different times (in mL/g).

Varieties	1 h	2 h	4 h	8 h	16 h	32 h	48 h
093	10.46 ± 0.21 a	16.35 ± 0.16 b	25.48 ± 0.1 c	45.59 ± 0.36 c	85.22 ± 0.65 c	109.6 ± 0.61 c	116.99 ± 0.96 c
137	12.77 ± 1.51 a	20.45 ± 1.39 a	32.7 ± 1.77 ab	62.63 ± 2.69 ab	94.98 ± 0.4 ab	116.3 ± 1.38 b	123.7 ± 1.47 b
231	12.42 ± 0.64 a	19.47 ± 0.84 ab	31.78 ± 1.12 ab	62.69 ± 1.99 ab	95.68 ± 1.95 ab	116.76 ± 1.85 b	124.85 ± 2.37 b
238	14.04 ± 0.66 a	20.86 ± 0.75 a	33.11 ± 1.06 ab	63.03 ± 2.02 ab	93.42 ± 1.39 b	116.07 ± 3.26 b	121.85 ± 2.66 bc
565	13.38 ± 0.8 a	19.9 ± 0.87 ab	29.54 ± 1.15 abc	54.01 ± 1.92 bc	90.48 ± 1.59 bc	115.37 ± 1.73 bc	123.41 ± 1.86 b
666	14.01 ± 1.43 a	21.21 ± 1.87 a	33.83 ± 3.37 a	66.42 ± 6.64 a	100.81 ± 4.9 ab	125.07 ± 2.46 a	132.97 ± 2.17 a
770	13.52 ± 0.72 a	19.83 ± 0.66 ab	30.16 ± 0.44 abc	56.44 ± 1.01 ab	91.03 ± 0.36 bc	113.63 ± 0.85 bc	120.77 ± 1.21 bc
811	12.48 ± 0.62 a	18.09 ± 0.75 ab	27.73 ± 0.78 bc	54.84 ± 1.99 bc	91.79 ± 1.29 bc	112.59 ± 1.23 bc	120.56 ± 1.4 bc
SEM	0.71	0.87	1.45	3.14	2.15	2.06	2.12
*p* value	0.201	0.069	0.017	0.03	0.005	0.002	0.001

Note: Different letters represent significant differences (*p* < 0.05) and three replicates were taken for each experiment. Total gas production was taken at each different time. All of the above ingredients are measured on a dry matter basis. Where h: hours

**Table 6:** Gas composition of silage quinoa lines.

Varieties	GP (mL/g)	CH_4_ (mL/g)	CO_2_ (mL/g)	O_2_, N_2_ (mL/g)
093	116.99 ± 0.95 c	8.59 ± 0.54 b	27.78 ± 1.81 cd	77.19 ± 1.99 d
137	123.7 ± 1.47 b	9.43 ± 0.18 b	32.19 ± 0.33 ab	78.38 ± 1.03 cd
231	124.85 ± 2.36 b	9.2 ± 0.5 b	29.8 ± 1.55 abc	82.06 ± 1.85 bcd
238	121.85 ± 2.65 bc	14.03 ± 0.83 a	32.93 ± 1.09 a	70.82 ± 2.79 e
565	123.41 ± 1.86 b	8.36 ± 0.23 b	27.03 ± 0.83 cd	84.55 ± 0.87 b
666	132.97 ± 2.17 a	9.19 ± 0.43 b	28.59 ± 0.41 bcd	90.76 ± 1.57 a
770	120.77 ± 1.2b c	7.99 ± 0.16 b	24.76 ± 0.97 de	83.74 ± 1.22 bc
811	120.56 ± 1.4 bc	8.17 ± 0.64 b	22.72 ± 1.22 e	85.17 ± 1.86 ab
SEM	1.06	10.6	0.8	1.3
*p* value	<0.01	<0.01	<0.01	<0.01

Note: Different letters represent significant differences (*p* < 0.05). Three replicates were taken for each experiment. GP is total gas production, and H_2_ was not detected or was negligible at less than 0.01%. All of the above ingredients are measured on a dry matter basis.

**Table 7:** Organic matter digestibility, metabolizable energy, and net energy for lactation of different lines of quinoa silage.

Varieties	OMD (%)	ME (MJ/kg DM)	NE_L_ (MJ/kg DM)
093	52.86 ± 0.21 cd	5.97 ± 0.03 c	3.12 ± 0.02 c
137	52.2 ± 0.34 d	6.12 ± 0.06 bc	3.2 ± 0.04 bc
231	54.49 ± 0.51 ab	6.19 ± 0.07 ab	3.3 ± 0.05 ab
238	54.51 ± 0.56 ab	6.11 ± 0.07 bc	3.23 ± 0.05 bc
565	53.92 ± 0.4 bc	6.18 ± 0.05 ab	3.27 ± 0.04 ab
666	55.59 ± 0.44 a	6.34 ± 0.06 a	3.4 ± 0.04 a
770	54.78 ± 0.17 ab	6.09 ± 0.03 bc	3.2 ± 0.02 bc
811	55.84 ± 0.32 a	6.06 ± 0.04 bc	3.2 ± 0.03 bc
SEM	0.27	0.03	0.02
*p* value	<0.01	<0.01	<0.01

Note: Different letters represent significant differences (*p* < 0.05), Three replicates were taken for each experiment. OMD is organic matter digestibility. ME is metabolizable energy, and NE_L_ is net energy for lactation.

## Data Availability

The original contributions presented in the study are included in the article, further inquiries can be directed to the corresponding author.

## References

[1] Misiukiewicz A., Gao M., Filipiak W., Cieslak A., Patra A.K., Szumacher-Strabel M. (2021). Review: Methanogens and methane production in the digestive systems of nonruminant farm animals. Animal.

[2] Khanna N., Lin J., Liu X., Wang W. (2024). An assessment of China’s methane mitigation potential and costs and uncertainties through 2060. Nat. Commun..

[3] Cardador M.J., Reyes-Palomo C., Díaz-Gaona C., Arce L., Rodríguez-Estévez V. (2022). Review of the Methodologies for Measurement of Greenhouse Gas Emissions in Livestock Farming: Pig Farms as a Case of Study. Crit. Rev. Anal. Chem..

[4] Li S., Sun Y., Guo T., Liu W., Tong X., Zhang Z., Sun J., Yang Y., Yang S., Li D. (2024). *Sargassum mcclurei* Mitigating Methane Emissions and Affecting Rumen Microbial Community in In Vitro Rumen Fermentation. Animals.

[5] Eugène M., Klumpp K., Sauvant D. (2021). Methane mitigating options with forages fed to ruminants. Grass Forage Sci..

[6] Ibrahim T.A., Hassen A., Apostolides Z. (2022). The Antimethanogenic Potentials of Plant Extracts: Their Yields and Phytochemical Compositions as Affected by Extractive Solvents. Plants.

[7] Lileikis T., Nainiene R., Bliznikas S., Uchockis V. (2023). Dietary Ruminant Enteric Methane Mitigation Strategies: Current Findings, Potential Risks and Applicability. Animals.

[8] Cobellis G., Trabalza-Marinucci M., Yu Z. (2016). Critical evaluation of essential oils as rumen modifiers in ruminant nutrition: A review. Sci. Total Environ..

[9] Ku-Vera J.C., Jimenez-Ocampo R., Valencia-Salazar S.S., Montoya-Flores M.D., Molina-Botero I.C., Arango J., Gomez-Bravo C.A., Aguilar-Perez C.F., Solorio-Sanchez F.J. (2020). Role of Secondary Plant Metabolites on Enteric Methane Mitigation in Ruminants. Front. Vet. Sci..

[10] Voronov S., Pleskachiov Y., Shitikova A., Zargar M., Abdelkader M. (2023). Diversity of the Biological and Proteinogenic Characteristics of Quinoa Genotypes as a Multi-Purpose Crop. Agronomy.

[11] Yilmaz Ş., Ertekin İ., Atiş İ. (2021). Forage yield and quality of quinoa (*Chenopodium quinoa* Willd.) genotypes harvested at different cutting stages under Mediterranean conditions. Turk. J. Field Crops.

[12] Villacres E., Quelal M., Galarza S., Iza D., Silva E. (2022). Nutritional Value and Bioactive Compounds of Leaves and Grains from Quinoa (*Chenopodium quinoa* Willd.). Plants.

[13] Agarwal N., Kolba N., Khen N., Even C., Turjeman S., Koren O., Tako E. (2022). Quinoa Soluble Fiber and Quercetin Alter the Composition of the Gut Microbiome and Improve Brush Border Membrane Morphology In Vivo (*Gallus gallus*). Nutrients.

[14] Ren G., Teng C., Fan X., Guo S., Zhao G., Zhang L., Liang Z., Qin P. (2023). Nutrient composition, functional activity and industrial applications of quinoa (*Chenopodium quinoa* Willd.). Food Chem..

[15] Kuljanabhagavad T., Wink M. (2009). Biological activities and chemistry of saponins from *Chenopodium quinoa* Willd. Phytochem. Rev..

[16] Alvar-Beltrán J., Dalla Marta A., Vivoli R., Verdi L., Orlandini S. (2022). Greenhouse Gas Emissions and Yield Production from an Organic and Conventional Fertilization on Quinoa. Agronomy.

[17] Zulkadir G., İdikut L. (2021). The impact of various sowing applications on the nutritional value of Quinoa Dry Herb. J. Food Process. Preserv..

[18] Ebeid H.M., Kholif A.E., El-Bordeny N., Chrenkova M., Mlynekova Z., Hansen H.H. (2022). Nutritive value of quinoa (*Chenopodium quinoa*) as a feed for ruminants: In sacco degradability and in vitro gas production. Environ. Sci. Pollut. Res. Int..

[19] Mustafa S., Riaz M.A., Masoud M.S., Qasim M., Riaz A. (2022). Impact of dietary inclusion of *Chenopodium quinoa* on growth performance and survival of Hubbard chicken. PLoS ONE.

[20] Marino R., Caroprese M., Annicchiarico G., Ciampi F., Ciliberti M.G., della Malva A., Santillo A., Sevi A., Albenzio M. (2018). Effect of Diet Supplementation with Quinoa Seed and/or Linseed on Immune Response, Productivity and Meat Quality in Merinos Derived Lambs. Animals.

[21] Jafari S., Ebrahimi M., Goh Y.M., Rajion M.A., Jahromi M.F., Al-Jumaili W.S. (2019). Manipulation of Rumen Fermentation and Methane Gas Production by Plant Secondary Metabolites (Saponin, Tannin and Essential Oil)—A Review of Ten-Year Studies. Ann. Anim. Sci..

[22] Elghandour M.M.Y., Vallejo L.H., Salem A.Z.M., Mellado M., Camacho L.M., Cipriano M., Olafadehan O.A., Olivares J., Rojas S. (2017). Moringa oleifera leaf meal as an environmental friendly protein source for ruminants: Biomethane and carbon dioxide production, and fermentation characteristics. J. Clean. Prod..

[23] Menke K.H., Steingass H. (1988). Estimation of the energetic feed value obtained from chemical analysis and in vitro gas productionusing rumen fluid. Anim. Res. Dev..

[24] Matra M., Suriyapha C., Dagaew G., Prachumchai R., Phupaboon S., Sommai S., Wanapat M. (2024). Advantageous effects of rumen-protected phytonutrients from tropical plant extracts on rumen fermentation efficiency and methane mitigation using in vitro fermentation technique. Anim. Biosci..

[25] (2017). Stationary Source Emission—Determination of Total Hydrocarbons, Methane and Nonmethane Hydrocarbons—Gas Chromatography.

[26] D’Souza G.M., Norris A.B., Tedeschi L.O. (2020). Evaluation of methane concentration sampling methods of gas produced from in vitro fermentation. J. Anim. Sci..

[27] Tonamo A., Tamir B., Goshu G. (2016). Assessment of Cattle Feed Resources; Chemical Composition and Digestibility of Major Feeds in Essera District, Southern Ethiopia. Sci. Technol. Arts Res. J..

[28] Gebremariam T., Belay S. (2021). Chemical Composition and Digestibility of Major Feed Resources in Tanqua-Abergelle District of Central Tigray, Northern Ethiopia. Sci. World J..

[29] Hare K.S., Lambert K., Chagas A.C., Watanabe D.H.M., Penner G.B. (2023). PSXII-30 Changes in Ruminal Digesta and Ruminal Ammonia-N Concentration with Differing Durations Feed Restriction for Sheep. J. Anim. Sci..

[30] Xiao F., Chen C., Gong W., Xiong Y., Zhou Y., Guo W., Li B., Wang Y. (2022). Trade-off between shade tolerance and chemical resistance of invasive *Phytolacca americana* under different light levels compared with its native and exotic non-invasive congeners. Environ. Exp. Bot..

[31] Zhou Y., Chen C., Xiong Y., Xiao F., Wang Y. (2023). Heavy metal induced resistance to herbivore of invasive plant: Implications from inter- and intraspecific comparisons. Front. Plant Sci..

[32] Sun J., Shi W., Wu Y., Ji J., Feng J., Zhao J., Shi X., Du C., Chen W., Liu J. (2021). Variations in Acorn Traits in Two Oak Species: *Quercus mongolica* Fisch. ex Ledeb. and *Quercus variabilis* Blume. Forests.

[33] Jiang Z., He J., Fang Y., Lin J., Liu S., Wu Y., Huang X. (2023). Effects of herbivore on seagrass, epiphyte and sediment carbon sequestration in tropical seagrass bed. Mar. Environ. Res..

[34] Ni B.B., Liu H., Wang Z.S., Zhang G.Y., Sang Z.Y., Liu J.J., He C.Y., Zhang J.G. (2024). A chromosome-scale genome of *Rhus chinensis* Mill. provides new insights into plant-insect interaction and gallotannins biosynthesis. Plant J..

[35] Olfaz M., Kilic U., Boga M., Abdi A.M. (2018). Determination of the In Vitro Gas Production and Potential Feed Value of Olive, Mulberry and Sour Orange Tree Leaves. Open Life Sci..

[36] He C., Li Q., Xiao H., Sun X., Gao Z., Cai Y., Zhao S. (2025). Effects of Mixing Ratio and Lactic Acid Bacteria Preparation on the Quality of Whole-Plant Quinoa and Whole-Plant Corn or Stevia Powder Mixed Silage. Microorganisms.

[37] Asher A., Galili S., Whitney T., Rubinovich L. (2020). The potential of quinoa (*Chenopodium quinoa*) cultivation in Israel as a dual-purpose crop for grain production and livestock feed. Sci. Hortic..

[38] Pazla R., Jamarun N., Agustin F., Arief A., Elihasridas E., Ramaiyulis R., Yanti G., Ardani L.R., Sucitra L.S., Ikhlas Z. (2024). Nutrition profile and rumen fermentation of *Tithonia diversifolia* fermented with *Lactobacillus bulgaricus* at different times and doses. J. Adv. Vet. Anim. Res..

[39] Dong Z., Li X., Fang D., Wang S., Li J., Dong D., Wang Y., Shao T. (2022). Effects of additives on the fermentation quality and bacterial community of silage prepared from fresh-cut whole-plant quinoa (*Chenopodium quinoa* willd.). Ital. J. Anim. Sci..

[40] Ertekin I., Atis I., Yilmaz S. (2023). The Effect of Cultivar and Stage of Growth on the Fermentation, Aerobic Stability and Nutritive Value of Ensiled Quinoa. J. Agric. Sci.-Tarim Bilim. Derg..

[41] Fang D., Dong Z.H., Wang D.L., Li B., Shi P.B.A., Yan J., Zhuang D.Y., Shao T., Wang W.Y., Gu M.F. (2022). Evaluating the fermentation quality and bacterial community of high-moisture whole-plant quinoa silage ensiled with different additives. J. Appl. Microbiol..

[42] Limon-Hernandez D., Rayas-Amor A.A., Garcia-Martinez A., Estrada-Flores J.G., Lopez M.N., Cruz Monterrosa R.G., Morales-Almaraz E. (2019). Chemical composition, in vitro gas production, methane production and fatty acid profile of canola silage (*Brassica napus*) with four levels of molasses. Trop. Anim. Health Prod..

[43] Suárez Mvz M.N.J.P., Escobar Mvz E.M.M.I., Molano Zoot E.M.C.E.R. (2019). Valor nutricional del ensilaje de forraje de quinua (*Chenopodium quinoa* willd) con adición de microorganismos eficientes. CES Med. Vet. Y Zootec..

[44] Patra A.K., Saxena J. (2009). Dietary phytochemicals as rumen modifiers: A review of the effects on microbial populations. Antonie Leeuwenhoek.

[45] Zhou X., Yue T., Wei Z., Yang L., Zhang L., Wu B. (2023). Evaluation of nutritional value, bioactivity and mineral content of quinoa bran in China and its potential use in the food industry. Curr. Res. Food Sci..

[46] Totakul P., Matra M., Sommai S., Viennasay B., Wanapat M. (2024). Combination effects of phytonutrient pellet and lemongrass (*Cymbopogon citratus*) powder on rumen fermentation efficiency and nutrient degradability using in vitro technique. Trop. Anim. Health Prod..

[47] Goel G., Makkar H.P.S., Becker K. (2008). Changes in microbial community structure, methanogenesis and rumen fermentation in response to saponin-rich fractions from different plant materials. J. Appl. Microbiol..

[48] Aderao G.N., Sahoo A., Bhatt R.S., Kumawat P.K., Soni L. (2018). In vitro rumen fermentation kinetics, metabolite production, methane and substrate degradability of polyphenol rich plant leaves and their component complete feed blocks. J. Anim. Sci. Technol..

[49] Zeru A.E., Hassen A., Apostolides Z., Tjelele J. (2022). Relationships Between Agronomic Traits of Moringa Accessions and In Vitro Gas Production Characteristics of a Test Feed Incubated with or Without Moringa Plant Leaf Extracts. Plants.

[50] Wang L., Gao H., Sun C., Huang L. (2022). Protective Application of Morus and Its Extracts in Animal Production. Animals.

[51] Hassan F.U., Arshad M.A., Li M., Rehman M.S., Loor J.J., Huang J. (2020). Potential of Mulberry Leaf Biomass and Its Flavonoids to Improve Production and Health in Ruminants: Mechanistic Insights and Prospects. Animals.

[52] Fiorentino S., Bellani L., Santin M., Castagna A., Echeverria M.C., Giorgetti L. (2025). Effects of Microalgae as Biostimulants on Plant Growth, Content of Antioxidant Molecules and Total Antioxidant Capacity in *Chenopodium quinoa* Exposed to Salt Stress. Plants.

[53] Ku-Vera J.C., Castelan-Ortega O.A., Galindo-Maldonado F.A., Arango J., Chirinda N., Jimenez-Ocampo R., Valencia-Salazar S.S., Flores-Santiago E.J., Montoya-Flores M.D., Molina-Botero I.C. (2020). Review: Strategies for enteric methane mitigation in cattle fed tropical forages. Animal.

[54] Kozlowska M., Cieslak A., Józwik A., El-Sherbiny M., Stochmal A., Oleszek W., Kowalczyk M., Filipiak W., Szumacher-Strabel M. (2020). The effect of total and individual alfalfa saponins on rumen methane production. J. Sci. Food Agric..

[55] Mahmoud A.E.M., Abbas M.S., Cieslak A., Szumacher-Strabel M. (2017). Evaluation of chemical composition and in vitro dry and organic matter digestibility of some forage plant species derived from Egyptian rangelands. J. Anim. Plant Sci..

[56] Lwin D.S., Williams A., Barber D.E., Benvenutti M.A., Williams B., Poppi D.P., Harper K.J., Watt L. (2022). Comparison of equations to predict the metabolisable energy content as applied to the vertical strata and plant parts of forage sorghum. Anim. Prod. Sci..

[57] Quintero-Anzueta S., Molina-Botero I.C., Ramirez-Navas J.S., Rao I., Chirinda N., Barahona-Rosales R., Moorby J., Arango J. (2021). Nutritional Evaluation of Tropical Forage Grass Alone and Grass-Legume Diets to Reduce In Vitro Methane Production. Front. Sustain. Food Syst..

[58] Pal K., Patra A.K., Sahoo A. (2015). Evaluation of feeds from tropical origin for in vitro methane production potential and rumen fermentation in vitro. Span. J. Agric. Res..

[59] Hariadi B.T., Santoso B. (2010). Evaluation of tropical plants containing tannin on in vitro methanogenesis and fermentation parameters using rumen fluid. J. Sci. Food Agric..

[60] Angeles-Mayorga Y., Cen-Cen E.R., Crosby-Galvan M.M., Ramirez-Bribiesca J.E., Candelaria-Martinez B., Sanchez-Villarreal A., Ramirez-Mella M. (2022). Foliage of Tropical Trees and Shrubs and Their Secondary Metabolites Modify In Vitro Ruminal Fermentation, Methane and Gas Production without a Tight Correlation with the Microbiota. Animals.

[61] Trotta R.J., Kreikemeier K.K., Foote S., McLeod K.R., Harmon D.L. (2023). Influence of Anti-Coccidial Compounds and Phytogenic Saponin Extracts on In Vitro and In Vivo Ruminal Fermentation and Methane Production of Cattle. Animals.

[62] Jensen R.H., Ronn M., Thorsteinsson M., Olijhoek D.W., Nielsen M.O., Norskov N.P. (2022). Untargeted Metabolomics Combined with Solid Phase Fractionation for Systematic Characterization of Bioactive Compounds in Hemp with Methane Mitigation Potential. Metabolites.

[63] Jayanegara A., Makkar H., Becker K. (2009). The use of principal component analysis in identifying and integrating variables related to forage quality and methane production. J. Indones. Trop. Anim. Agric..

